# Antibiotic use attributable to RSV infections during infancy—an international prospective birth cohort study

**DOI:** 10.1093/jac/dkaf123

**Published:** 2025-05-09

**Authors:** Sarah F Hak, Roderick P Venekamp, Marie-Noëlle Billard, Daniela Cianci, Marlies A Van Houten, Andrew J Pollard, Terho Heikkinen, Steve Cunningham, Margaret Millar, Federico Martinon-Torres, Ana Dacosta-Urbieta, Louis J Bont, Joanne G Wildenbeest, PROMISE Investigators, Sarah Hak, Roderick Venekamp, Joanne Wildenbeest, Marie-Noëlle Billard, Marlies van Houten, Louis Bont, Andrew Pollard, Ana Dacosta-Urbieta, Federico Martinón-Torres, Terho Heikkinen, Steve Cunningham, Harish Nair, Margaret Miller, Peter Openshaw, Philippe Beutels, Hannah Nohynek, Anne Teirlinck, Christos Baliatsas, Leyla Kragten, Thea Fisher, Carlo Giaquinto, Javier Diez-Domingo, Rafael Mikolajczyk, Jim Jamaniak, Elizabeth Begier, Jenny Hendri, Rolf Kramer, Veena Kumar, Bahar Ahani, Eva Molero

**Affiliations:** Department of Paediatric Infectious Diseases and Immunology, Wilhelmina Children’s Hospital/University Medical Center Utrecht, Utrecht, The Netherlands; Julius Center for Health Sciences and Primary Care, University Medical Center Utrecht, Utrecht, The Netherlands; Department of Paediatric Infectious Diseases and Immunology, Wilhelmina Children’s Hospital/University Medical Center Utrecht, Utrecht, The Netherlands; Department of Data Science and Biostatistics, University Medical Center Utrecht, Utrecht, The Netherlands; Department of Paediatrics, Spaarne Gasthuis Academy, Hoofddorp and Haarlem, The Netherlands; Department of Paediatrics, Oxford Vaccine Group, University of Oxford, and the NIHR Oxford, Biomedical Research Centre, Oxford, UK; Department of Pediatrics, University of Turku and Turku University Hospital, Turku, Finland; Centre for Inflammation Research, University of Edinburgh, Edinburgh, UK; Children’s Clinical Research Facility, NHS Lothian, Edinburgh, UK; Translational Pediatrics and Infectious Diseases, Pediatrics Department, Hospital Clínico Universitario de Santiago de Compostela, Santiago de Compostela, Spain; Genetics, Vaccines and Infections Research Group (GENVIP), Instituto de Investigación Sanitaria de Santiago, University of Santiago de Compostela, Santiago de Compostela, Spain; Centro de Investigación Biomédica en Red de Enfermedades Respiratorias (CIBERES), Instituto de Salud Carlos III, Madrid, Spain; Translational Pediatrics and Infectious Diseases, Pediatrics Department, Hospital Clínico Universitario de Santiago de Compostela, Santiago de Compostela, Spain; Genetics, Vaccines and Infections Research Group (GENVIP), Instituto de Investigación Sanitaria de Santiago, University of Santiago de Compostela, Santiago de Compostela, Spain; Centro de Investigación Biomédica en Red de Enfermedades Respiratorias (CIBERES), Instituto de Salud Carlos III, Madrid, Spain; Department of Paediatric Infectious Diseases and Immunology, Wilhelmina Children’s Hospital/University Medical Center Utrecht, Utrecht, The Netherlands; Department of Paediatric Infectious Diseases and Immunology, Wilhelmina Children’s Hospital/University Medical Center Utrecht, Utrecht, The Netherlands

## Abstract

**Background:**

Early-life antibiotic use impacts microbiome composition and contributes to the emergence of antimicrobial resistance. Despite respiratory syncytial virus (RSV) being a leading cause of acute respiratory infections (ARI), accurate estimates of antibiotic use attributable to RSV are lacking.

**Objectives:**

To assess RSV-associated antibiotic use during the first year of life.

**Patients and methods:**

The RESCEU birth cohort study followed healthy term infants, born (*n* = 9154) between 1 July 2017 and 31 July 2020 from five European countries, to identify RSV-ARI hospitalizations during infancy. In a nested cohort (*n* = 993), we performed active RSV surveillance by collecting nasal swabs in case of ARI symptoms during RSV seasons (October–April). Antibiotic use during hospitalization was identified through chart review, while outpatient data were collected via parental questionnaires.

**Results:**

In the total cohort, antibiotics were used in 22.8% of RSV hospitalizations (33/145) and 62.5% of RSV intensive care admissions (5/8). In the nested cohort, antibiotics were used in 5.2% of any-severity RSV-ARI (13/250) and 9.9% of medically attended RSV-ARI (13/131). This results in an estimated incidence of 1.3% (95%CI: 0.8–2.0) of healthy term infants receiving ≥1 course of antibiotics associated with RSV infection in their first year, with an incidence rate of 1.1 RSV-associated antibiotic prescriptions per 1000 infant-months (95%CI: 0.6–1.9). As such, RSV accounts for 22.9% of antibiotic prescriptions for ARI during RSV seasons.

**Conclusions:**

One in 77 healthy term infants receives antibiotics during RSV infection before their first birthday. Real-world evidence is needed to establish the impact of RSV immunization on antibiotic use during infancy.

**Clinical Trials Registration:**

NCT03627572.

## Introduction

Acute respiratory infections (ARI) are the most common reason for primary care visits in young children and a major driver of antibiotic prescriptions, despite a predominantly viral aetiology.^1,2^ Early-life antibiotic use not only contributes to the emergence of antimicrobial resistance (AMR),^3^ but is also implicated in healthy maturation of the gut microbiota,^4,5^ and is associated with increased risk of celiac and inflammatory bowel disease,^6,7^ asthma^8^ and obesity.^9^ Concerted efforts to reduce childhood antibiotic use are therefore needed.

Evidence is accumulating that respiratory syncytial virus (RSV) accounts for a substantial proportion of ARI during early life: one in every four healthy infants experiences at least one symptomatic RSV infection during their first year of life,^10^ and RSV may account for up to 40% of ARI-related primary care visits in infants during RSV seasons.^11^ The introduction of novel maternal RSV vaccines and long-acting monoclonal antibodies hold promise to reduce severe RSV infections in infants and associated antibiotic use.^12–14^

While the community-burden of RSV is increasingly studied, prospective data on RSV-associated antibiotic use in the community are scarce [see the Supplementary literary review for a review of available literature and Tables S1–S4 (available as Supplementary data at *JAC* Online]).^15,16^ Previous studies based on national surveillance data provided estimates of community-based antibiotic prescribing for RSV, but these estimates rely on modelling rather than real-world data,^17,18^ due to absence of routine RSV testing in outpatient settings. Accurate data on RSV-associated antibiotic use are pivotal to understand the full impact of widespread introduction of RSV immunization, as well as to inform AMR prevention strategies.

We, therefore, determined the incidence of RSV-associated antibiotic use in a birth cohort of healthy term-born infants, and we quantified the fraction of antibiotic use for ARI that can be attributed to RSV during the RSV season. Additionally, we explored whether RSV hospitalization was linked to increased first-year antibiotic use for ARI not related to the hospitalization itself.

## Patients and methods

### Study participants

This study is part of the RESCEU prospective birth cohort study (ClinicalTrials.gov identifier NCT03756766), for which methods have been previously described.^19^ Briefly, healthy term-born infants from five European countries (Spain, Finland, England, Scotland and the Netherlands) were enrolled at birth between 1 July 2017 and 31 July 2020. Infants were considered healthy term-born if born after at least 37 weeks of gestation and showed no sign of significant cardiovascular, respiratory, renal, gastrointestinal, haematological, neurological, endocrine, immunological, musculoskeletal, oncological or congenital disorders. Written or electronic informed consent was obtained from the parents of all study participants. The study received approval from the institutional review boards in all five countries.

### Study design and procedures

In the total birth cohort, passive RSV surveillance was performed through parental questionnaires at the age of 1 year. This questionnaire was used to screen for hospitalization for ARI during the first year of life. In case of hospitalization for ARI, hospital records were retrospectively assessed for RSV test results and the use of antibiotics during admission. In case parents did not complete the 1-year questionnaire, records of participating hospitals were screened to assess whether their infant was hospitalized for ARI in the first year of life. The 1-year questionnaire also inquired whether the infant had used any antibiotics for ARI in the first year of life.

At enrolment, participants of the total birth cohort were invited to also participate in a nested cohort (referred to as active surveillance cohort). Each site was instructed to recruit 15–20 participants per week for the total cohort, including two participants in the active surveillance cohort. Participation in the active surveillance cohort was on voluntary basis and thus a convenience sample of the total birth cohort. This subset of infants was actively followed up throughout the first year of life. During the RSV season, which was defined as 1 October to 1 May (or longer if RSV was still circulating), parents were contacted weekly to report ARI symptoms in their child. On notification of an ARI, a home visit was scheduled within 72 hours to obtain a nasal swab for RSV testing. An ARI episode was defined as the onset or worsening of any of the following symptoms for at least 1 day: runny or blocked nose, coughing, wheezing or dyspnoea. ARI episodes were associated with RSV if a point-of-care test (or in-house polymerase chain reaction test was positive for RSV. Samples taken >10 days after ARI symptom onset were excluded from analysis. For every ARI, parents completed a daily diary of symptoms for 14 days from ARI symptoms onset. On day 15, parents completed a final questionnaire on healthcare usage, antibiotic use and any remaining symptoms.

### Outcomes and statistical analysis

Demographics and clinical characteristics are summarized by frequency (%) for categorical variables and mean (standard deviation) or median (IQR) for continuous variables. We present the proportion (%) of documented antibiotic use for ARI hospitalizations in the total cohort, and for all ARI in the active surveillance cohorts, stratified by healthcare setting. Proportions were compared between RSV-positive and -negative ARI and hospitalizations using *χ*^2^ or Fisher’s exact tests.

In the active surveillance cohort, we report all-cause ARI- and RSV-associated antibiotic use, as incidence proportion (i.e. the proportion of infants experiencing the event at least once during the first year of life) and incidence rates per 1000 infant-months (number of antibiotic prescriptions per 1000 infant-months of follow-up). We used incidence rates in addition to incidence proportion to account for participants experiencing outcomes more than once, and for possible variation in follow-up time due to early dropouts. We calculated the fraction of antibiotic use attributable to RSV, by dividing the incidence rate of RSV-associated antibiotic use by the incidence rate of ARI-associated antibiotic use.

Regarding missing data, a two-step procedure multiple imputation procedure was applied. First, invalid or missing RSV test results and missing observations regarding medical attendance were imputed based on site, sex, age, and meteorological season at time of ARI.^10^ Second, missing data on hospitalizations and antibiotic use was imputed. In case of missing data on antibiotic use during non-medically attended ARI, we assumed no antibiotic use. Conversely, when antibiotic use was documented during non-medically attended ARIs (*n* = 5), we inferred medical attendance, since antibiotics are only available by doctor prescription in the participating countries. For medically attended ARI, we subsequently imputed any missing data on hospitalization and antibiotic use, based on site, sex, age and meteorological season at time of ARI, RSV and medical attendance status. Imputation yielded 10 complete datasets. Estimates for proportions and incidence rates were obtained for the different complete datasets. For each imputed dataset, use of antibiotics between non-RSV and RSV-ARI episodes were compared using generalized estimating equations with an exchangeable correlation structure to account for repeated episodes of the same child. Multiple imputation was performed using the MICE package and based on a logistic regression model.^20^ Pooled estimates, 95% Cis and *P* values were subsequently derived by applying Rubin’s rules, using the pool function from the MICE package.

As an exploratory analysis, we report the proportion of infants in the total cohort who received antibiotics for ARI during the first year of life, as reported in the year-1 questionnaire. We compare this proportion between infants hospitalized with RSV-ARI, those hospitalized with RSV-negative ARI and those not hospitalized with ARI, excluding antibiotics administered during hospitalization, with comparisons made using the *χ*² test.

Statistical analyses were conducted using SPSS (version 28) and R statistical software (version 4.3.1). A *P* value ≤0.05 was deemed statistically significant.

## Results

### Study population

**Figure 1.**
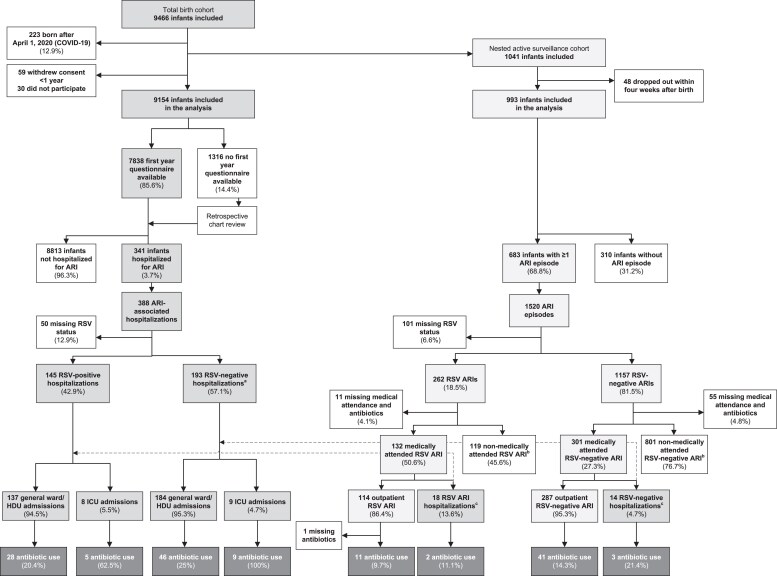
Flowchart of study participants and reported ARI-associated antibiotic use. HDU, high-dependency unit. ^a^For 21 ARI-associated hospitalizations with a missing RSV test result, RSV status was assumed negative since the hospitalization occurred outside of the RSV season. ^b^Antibiotic use was reported for one RSV-positive ARI and four RSV-negative ARIs for which no medical consultation was reported. For these episodes, we inferred outpatient medical consultation, since antibiotics are only available by doctor prescription in the participating countries. ^c^Hospitalizations of infants in the active cohort are also included in the data for the total cohort.

Between 1 July 2017 and 31 July 2020, 9466 healthy term infants were recruited at birth, of whom 9154 (96.7%) were included in the primary analysis (Figure 1). Because of the COVID-19 pandemic, 223 infants born after 1 April 2020 were excluded from all analyses as RSV was not circulating during their first year of life. Key demographic and clinical characteristics of the total study population are listed in Table 1. Characteristics by country are available in our previous publication on the RESCEU birth cohort.^10^ Within the total cohort, there were relatively few participants from Spain (12%), whereas in the active cohort the participants’ country of origin was evenly distributed among the five countries represented. The rates of caesarean section deliveries and antibiotic administration within 72 hours after birth were comparable between the two cohorts (29% versus 30%, and 4% versus 3%, in the total and active cohorts, respectively).

**Table 1.** Key demographics of study population

	Total cohort (*n* = 9154)	Active surveillance cohort (*n* = 993)
Country		
Scotland	2130 (23%)	203 (20%)
England	1972 (22%)	198 (20%)
Spain	1080 (12%)	205 (21%)
Finland	2093 (23%)	200 (20%)
The Netherlands	1879 (21%)	187 (19%)
Male sex	4725/9116 (52%)	512/992 (52%)
Multiple birth	197/9105 (2%)	38/990 (4%)
Caesarean delivery	2607/9110 (29%)	296/992 (30%)
Antibiotics <72 h post-partum	346/9089 (4%)	25/992 (3%)
Intention to breastfeed	7472/9035 (83%)	860/990 (87%)
Family: any siblings	4432/9099 (49%)	504/992 (51%)
Family history of		
Asthma	2307/9042 (26%)	241/985 (25%)
Eczema	2863/9036 (32%)	315/986 (32%)
Allergy/hay fever	5040/9028 (56%)	588/988 (60%)
Smoking within family	861/9099 (9%)	137/989 (14%)
Smoking in the house	98/9037 (1%)	12/989 (1%)
Ethnic origin of the mother		
Northwest Europe	6592/9037 (73%)	656/990 (66%)
Southern Europe	1122/9037 (12%)	200/990 (20%)
Other	1323/9037 (15%)	134/990 (14%)
Ethnic origin of the father		
Northwest Europe	6567/9006 (73%)	677/987 (69%)
Southern Europe	1114/9006 (12%)	193/987 (20%)
Other	1325/9006 (15%)	117/987 (11%)
Highest level of education of the mother		
Secondary or vocational school	3364/9039 (37%)	287/990 (29%)
University of (applied) sciences	5554/9039 (61%)	692/990 (70%)
Highest level of education of the father		
Secondary or vocational school	4316/8944 (48%)	421/984 (43%)
University of (applied) sciences	4399/8944 (49%)	543/984 (55%)

Demographics by country are available in our previous publication on the RESCEU birth cohort.^10^

### Number of hospitalizations, ARIs, medical attendance and antibiotic use

In the total cohort, we recorded 388 ARI-associated hospitalizations, of which 145 were RSV-positive, 193 were RSV-negative and for 50 no RSV test result was available. Antibiotics were prescribed in 88/388 (22.7%) of all ARI-associated hospitalizations, 33/145 (22.8%) of RSV-positive and 55/193 (28.5%) of RSV-negative hospitalizations (*P *= 0.23, Figure 1, Table 2). Among RSV-positive infants admitted to the intensive care unit (ICU), 5/8 (62.5%) received antibiotics.

**Table 2.** Antibiotic use in RSV-positive versus RSV-negative ARI episodes

	RSV-positive ARI		RSV-negative ARI		*P* value	
	Before imputation	After imputation	Before imputation	After imputation	Before imputation	After imputation^c^
Hospitalizations (total cohort)						
Antibiotic use						
All hospitalizations	22.8% (33/145)	23.2%	28.5% (55/193)	27.8%	0.23	0.33^c^
General ward/HDU	20.4% (28/137)	21.3%	25.0% (46/184)	24.7%	0.34	0.46^c^
Intensive care	62.5% (5/8)	58.1%	100% (9/9)	95.7%	0.08	0.21^c^
By country						
England	32.1% (9/28)	30.2%	31.8% (15/22)	26.4%	0.98	0.72^c^
Scotland	8.3% (4/48)	10.2%	23.9% (16/67)	25.2%	0.03	0.06^c^
Spain	40.0% (15/25)	39.6%	48.3% (13/29)	47.7%	0.54	0.48^c^
Finland	38.1% (8/21)	36.5%	25.0% (7/28)	24.1%	0.33	0.35^c^
The Netherlands	8.7% (2/23)	9.0%	23.4% (11/47)	23.0%	0.20	0.16^c^
Type of antibiotics						
Amoxicillin/clavulanic acid	12.1% (4/33)	15.2%	14.5% (8/55)	16.5%	1.00	0.87^e,f^
Amoxicillin	63.6% (21/33)	59.7%	36.4% (20/55)	35.4%	0.01	0.03^e,f^
Penicillin	2/33 (6.1%)	5.1%	7.3% 4/55	6.6%	1.00	0.75^e,f^
Gentamicin	0/33 (0.0%)	0.0%	9.1% (5/55)	8.3%	0.15	0.13^d^
Macrolide	1/33 (3.0%)	8.6%	9.1% (5/55)	10.9%	0.40	0.73^e,f^
Cephalosporins	8/33 (24.2%)	21.8%	28/55 (50.9%)	46.9%	0.01	0.02^e,f^
ARI (active surveillance cohort)						
Antibiotic use						
All ARI episodes	5.2% (13/250)	4.9%	4.0% (44/1102)	3.6%	0.39	0.31^c^
Medically attended^a,b^	9.9% (13/131)	9.5%	14.6% (41/301)	13.1%	0.19	0.28^c^
Outpatient	9.7% (11/113)	8.8%	14.3% (41/287)	12.9%	0.22	0.23^c^
Hospitalization^b^	11.1% (2/18)	13.7%	21.4% (3/14)	15.5%	0.63	0.83^c^
Type of antibiotics						
Augmentin	30.8% (4/13)	31.2%	14.3% (6/42)	11.9%	0.22	0.12^e,f^
Amoxicillin	69.2% (9/13)	62.5%	57.1% (24/42)	57.1%	0.44	0.74^e,f^
Penicillin	0.0% (0/13)	1.6%	2.4% (1/42)	6.7%	1.00	0.57^d,f^
Macrolide	7.7% (1/13)	7.8%	4.8% (2/42)	4.8%	0.56	0.68^e,f^
Cephalosporins	0.0% (0/13)	0.0%	7.1% (3/42)	4.8%	1.00	0.54^d,f^
Fluoroquinolone	0.0% (0/13)	0.0%	2.4% (1/42)	2.4%	1.00	0.71^d,f^
Trimethoprim	0.0% (0/13)	0.0%	7.1% (3/42)	4.8%	1.00	0.54^d,f^

GP, general practitioner; HDU, high-dependency unit.

^a^In case of medical attendance, ARI episode was categorized by the highest level of attended care.

^b^Hospitalizations reported in the active surveillance cohort are also included in the total cohort.

^c^ *P* values from a generalized estimating equation model with antibiotic use as outcome and RSV status as predictor and an exchangeable correlation structure pooled using Rubin’s rules.

^d^ *P* values from a Bayesian logistic regression model with antibiotic use as outcome and RSV status as predictor (to overcome separation with standard logistic regression).

^e^ *P* values from a logistic regression analysis (clustering with subjects was ignored here because only very few subjects had multiple ARI episodes with antibiotic use, namely 3/97 in passive and 2/53 in active cohort).

^f^The type of antibiotics was not imputed and therefore only those subjects with recorded antibiotic use were included when comparing the types of antibiotics between the subgroups defined by RSV status.

In the active surveillance cohort, we recorded 1520 ARIs in 683 infants, of which 262 were RSV-positive ARIs, 1157 were RSV-negative ARIs and 101 ARIs had missing RSV test results (Figure 1). Data on antibiotic use were missing for 92 ARIs. Overall, antibiotic use was reported in 57 out of 1428 ARIs with available data (4.0%) (Figure 1, Table 2). For RSV-positive ARIs, antibiotics were prescribed in 13 out of 250 (5.2%) episodes, of which 11 prescriptions were in outpatient settings and two were during hospitalization. For RSV-negative ARIs, antibiotics were prescribed in 44 out of 1102 (4.1%) episodes, of which 41 prescriptions were in outpatient settings and three were during hospitalization.

The proportion of ARI for which antibiotics were prescribed did not differ between all RSV-positive and -negative ARI (5.2% versus 4.1%, *P = *0.39*),* nor between medically attended RSV-positive and -negative ARI only (9.9% versus 14.6%, *P *= 0.19) (Table 2). Antibiotic type did not differ between RSV-positive and -negative ARIs, with amoxicillin being the most frequently prescribed in both groups (69.2% versus 57.1%, *P *= 0.44).

For both the total and active surveillance cohort, proportional antibiotic use during ARI episodes was similar after the imputation of missing data (Table 2). Country-specific data on RSV-associated antibiotic use are presented in the Supplementary data.

### Incidence of ARI-associated antibiotic use and RSV-attributable fraction

After imputing missing data, the incidence proportion of any ARI-associated antibiotic use during RSV seasons was estimated at 5.3% (95%CI: 4.2–6.7), with an incidence rate of 4.8 antibiotic prescriptions per 1000 infant-months (95%CI: 3.6–6.2) (Table 3). The incidence proportion of RSV-associated antibiotic use was 1.3% (95%CI: 0.8–2.0), with an incidence rate of 1.1 prescriptions per 1000 infant-months (95%CI: 0.6–1.9). The RSV-attributable fraction of ARI-associated antibiotic prescriptions during RSV seasons was 22.9%.

**Table 3.** Incidence proportion and rates of RSV-associated antibiotic use during RSV season (active surveillance cohort)

	After imputation^a,b^	Before imputation	Cohort size or person-time	Number of infants with ARI episodes (observed)	Number of infants/episodes with antibiotic prescriptions (observed)
All ARI during RSV season					
Incidence proportion^c^	5.3% (4.2–6.7)	5.1% (3.9–6.7)	993 infants	683 infants	51 infants
Incidence rate per 1000 infant-months^d^	4.8 (3.6–6.2)	4.9 (3.8–6.3)	11 728 infant-months	1520 episodes	57 episodes
RSV-positive ARI					
Incidence proportion^c^	1.3% (0.8–2.0)	1.3% (0.7–2.2)	993 infants	249 infants	13 infants
Incidence rate per 1000 infant-months^d^	1.1 (0.6–1.9)	1.1 (0.6–1.9)	11 728 infant-months	262 episodes	13 episodes
RSV-negative ARI					
Incidence proportion^c^	4.1% (3.2–5.3)	4.0% (3.0–5.4)	993 infants	411 infants	40 infants
Incidence rate per 1000 infant-months^d^	3.7 (2.7–5.0)	3.8 (2.8–5.0)	11 728 infant-months	1157 episodes	44 episodes
RSV-attributable fraction of ARI-associated antibiotic use during RSV season					
	22.9%	23.4%			

^a^Missing RSV and medical attendance status have previously been imputed using multiple imputation based on site, sex, age and meteorological season at time of hospitalization or ARI.^10^ We subsequently imputed any missing data on antibiotic use, based on the same predictors to which RSV and medical attendance status were previously added.

^b^Outcomes that required imputations included: 50 hospitalizations with missing RSV status, 166 ARI episodes with missing RSV status or missing medical attendance status, 101 ARI episodes with missing RSV status and 92 ARI episodes with missing antibiotic use status.

^c^Incidence as proportion of infants experiencing the event at least once during their first year of life.

^d^Incidence rate as number of events per 1000 infant-months of follow-up.

### RSV hospitalization and ARI-associated antibiotic use in first year of life

Based on the 1-year questionnaire, infants who had been hospitalized for RSV more often reported ARI-associated antibiotic use during the first year of life, excluding antibiotics used during the RSV hospitalization itself, compared to infants not hospitalized for RSV or other ARI (28.7% versus 15.2%, *P *< 0.001). First-year ARI-associated antibiotic use did not differ between infants hospitalized with RSV and those hospitalized with other ARIs (28.7% versus 38.5%%, *P *= 0.11) (Table 4).

## Discussion

We previously reported that one-quarter (26.2%) of healthy term infants develop a symptomatic RSV infection in the first year of life.^10^ With this study, we show that antibiotics are prescribed during 5.2% of all RSV ARIs, 9.9% of RSV medically attended ARIs and 22.8% of RSV hospitalizations. This translates to 1 in 77 healthy term infants (1.3%) having received at least one course of antibiotics due to RSV before their first birthday. As such, RSV accounts for nearly one-quarter of ARI-associated antibiotic prescriptions (22.9%) during the RSV season. Extrapolating to the EU birth cohort,^21^ this implies ∼50 000 RSV-associated antibiotic prescriptions in infants annually.

Current literature on RSV-associated antibiotic use is predominantly focused on hospitalized populations, with reported rates varying widely: 14%–51% during RSV hospitalization and 82%–88% during RSV ICU admissions (see the Supplementary data for a detailed summary of available literature). However, these studies were retrospective, were conducted exclusively in tertiary hospitals with generally more severely ill children or also included preterm infants and those with comorbidities, who are more likely to receive antibiotics due to increased risk of complications.^22^ To our knowledge, our study is the first to prospectively assess antibiotic use during RSV hospitalization in otherwise healthy term infants, showing that, even in this low-risk group, nearly one-quarter are treated with antibiotics.

Owing to the absence of routine RSV testing, data on RSV-associated antibiotic prescribing in outpatient settings are limited. Two prospective cohort studies provide rates of antibiotic use during laboratory-confirmed RSV infections in primary care, reporting prescription rates of 26.3% among children aged <2 years with RSV bronchiolitis in France and 15.2% among infants with RSV-ARI across five European countries, respectively.^11,23^

In contrast to the aforementioned studies, the major strength of this birth cohort study lies in its longitudinal, community-based design. This approach allowed tracking of RSV-associated antibiotic use across all levels of care (outpatient, secondary and tertiary care) over time, rather than providing only cross-sectional rates in specific healthcare settings. Three previous birth cohort studies, all from Finland, also report on RSV-associated antibiotic use. Thomas *et al.* showed that nearly one-third (33%) of healthy infants developed an RSV infection in the first year, with 71% of RSV infections treated with antibiotics.^15^ This implies that ∼25% of all infants will receive ≥1 course of antibiotics due to RSV by their first birthday, which is ∼20-fold higher than our study. This is partially due to the higher overall incidence of RSV infections (33% versus 26% in our cohort), but mainly results from the high antibiotic prescription rate. The authors propose the high incidence of acute otitis media (AOM) in RSV-infected infants (77%) as a major driver of antibiotic prescription. Heikkinen *et al.* similarly report high rates of AOM and antibiotic treatment (both 91%) among RSV-infected infants, although based on a small sample size.^24^ Of note, both studies required infants to visit the clinic for RSV testing and examination in case of ARI symptoms, even if symptoms may normally not warrant doctor consultation, potentially resulting in more RSV-associated AOM diagnoses and antibiotic prescribing. Toivonen *et al.* reported lower rates of AOM (31%) and antibiotic use (35%) in RSV-positive children aged <2 years.^16^ This translated to 16 RSV-associated antibiotic treatments per 100 children/year, accounting for 30% of antibiotic treatments for ARI during RSV seasons.

Population-based modelling studies provide further evidence of considerable antibiotic use attributable to RSV, by linking national viral surveillance to antibiotic dispensing data. In Scottish infants, it was estimated that RSV accounts for 5.2% of all primary care antibiotic prescriptions, with estimated ∼2.3 RSV-associated antibiotic prescriptions per 1000 infant-months.^17^ In the UK, it was estimated that 8.3% of children aged <6 months and 11.9% of those aged 6–23 months receive antibiotics due to RSV infection annually. As such, RSV would account for 19.7% of antibiotics prescribed among infants aged <6 months and 14.6% in those aged 6–23 months in primary care.^18^

Considering the aforementioned studies, the incidence of RSV-associated antibiotic use in our study appears comparatively low, primarily due to lower antibiotic prescribing rates in outpatient settings. One reason could be that our cohort consisted of healthy term infants only. Another explanation might be the availability of point-of-care RSV test results to parents in Spain, England and the Netherlands, which they could share with healthcare providers. This information may have affected outpatient antibiotic prescribing rates for ARI episodes,^25^ given that viral testing is usually unavailable in these settings, unlike in hospitals. Furthermore, antibiotics prescribed for RSV-associated AOM may not have been fully captured by our questionnaire, as parents may not have recognized this antibiotic use as related to the respiratory infection. Last, even within Europe, the incidence of RSV-associated antibiotic use may be highly country-specific, as also observed in our literature review (Supplementary data), probably reflecting differences in local prescribing practices.^26^ Our study suggests antibiotic prescribing was, indeed, highest in Finland among the participating countries. These variations highlight the potential of antimicrobial stewardship efforts aimed at standardizing practices across countries to reduce overprescribing. However, this study was not sufficiently powered for cross-country comparisons, and based on the limited number of infants who used antibiotics, these country-specific data should be interpreted with caution.

RSV may contribute to antibiotic use even beyond the acute infection phase. Our study found that infants hospitalized for RSV were nearly twice as likely to use antibiotics for ARI in their first year, excluding antibiotics during hospitalization itself. A potential explanation is that a child may be more susceptible to bacterial respiratory infections after severe RSV infection. Notably, increased first-year antibiotic use was observed for RSV-negative ARI hospitalizations as well, suggesting a non-RSV-specific effect. Also, we could not specify whether ARI-associated antibiotics were used before or after RSV hospitalization. Nonetheless, several other studies reported an increase in bacterial respiratory infections following RSV infection, including AOM, tonsillitis or pneumonia, and related antibiotic use.^27–31^ To illustrate, Spanish infants had a 4-fold increase in antibiotics use (10.3% versus 3%) in the 3 months after RSV admission and a 2-fold increase (18.7% versus 10.2%) in the following 9 months. Whether RSV infection is truly causally related to longer-term respiratory morbidity, or rather a marker of shared genetic predisposition, remains under debate.^32,33^

Additional limitations of our study should be acknowledged. First, we focused on detecting RSV infections and associated antibiotic use, therefore active ARI surveillance was conducted only during the RSV season (October–April). As a consequence, our reporting on ARI-associated antibiotic use is restricted to this period. Second, the COVID-19 pandemic affected RSV incidence in 2020. The 2019–2020 RSV season had mostly ended when the COVID-19 pandemic began, except in Finland, where it typically runs into late spring. This could have led to lower incidence of RSV infections and associated antibiotic use. However, we expect the impact to be minimal, since participants born after 1 April 2020 were excluded due to the absence of RSV circulation in their first year, and follow-up time after 1 November for the remaining infants represented <3% of total follow-time of the cohort. Third, information regarding the specific indications for antibiotic prescribing, such as suspected bacterial (co)infections or AOM was lacking. Therefore, we cannot elaborate on the appropriateness of antibiotic prescriptions. Nonetheless, we assume that antibiotics would not have been prescribed if no RSV infection occurred in this cohort without comorbidities. Fourth, the report of any ARI-associated antibiotic use during the first year of life, as collected via the year-1 questionnaire, may be subject to recall bias, with parents potentially misremembering antibiotic use. This limitation only applies to antibiotic use for outpatient ARI in the total cohort, as antibiotic use during ARI hospitalization was assessed through hospital chart review. Finally, we did not test for viral coinfections, yet these are probably prevalent.^34^ Although we cannot definitively establish RSV as the causal pathogen of ARI symptoms and associated antibiotic use, RSV detection is strongly correlated with symptomatic ARI.^35^

Recently, a maternal RSV vaccine and long-acting monoclonal antibody (nirsevimab) have been market-approved and are currently being implemented in immunization programmes across countries.^13,14^ Early real-world findings indicate that nirsevimab reduces the risk of RSV-related hospitalizations by >80% during the first 3 months after administration.^36^ In addition, by mitigating RSV infections, for which antibiotics are often wrongly prescribed, as well as secondary bacterial infections requiring antibiotics, RSV immunization has the potential to indirectly reduce both inappropriate and appropriate antibiotic use, as observed after implementation of influenza vaccination.^37–39^ A recent phase III trial showed a 12.9% decrease in all-cause antibiotic use during the first three months among infants whose mothers had received a maternal RSV vaccine.^12^ In the nirsevimab trial, antibiotic use in 150 days of follow-up was reduced by 23.6% in the intervention group, resulting in 8.2 antibiotic courses averted per 100 infants.^40^ These findings imply RSV is a major contributor to antibiotic prescribing during infancy, and that this is preventable by immunization.

In summary, this study demonstrates that RSV infection contributes significantly to antibiotic use in healthy term-born infants. Understanding the effects of RSV disease prevention on antibiotic use and AMR, along with its other potential secondary effects,^38^ is imperative for assessing the full impact of novel RSV immunizations. Real-world evidence following the introduction of RSV immunizations is needed to demonstrate whether RSV disease prevention can indeed reduce antibiotic use during infancy.

## Supplementary Material

dkaf123_Supplementary_Data

## Transparency declarations

S.H., R.P.V., D.C., M.M. and M.B. report no potential conflict of interest. M.A.V.H. has received honoraria for participation in advisory boards from GSK, Pfizer, Sanofi and Moderna and acted as PI in randomized clinical trials for Pfizer, with all fees paid to the Spaarne Gasthuis. T.H. has received honoraria for lectures or participation in advisory boards or data monitoring committees from Sanofi, MSD, Pfizer, Enanta, Moderna and Shionogi. A.J.P. is part of the RESCEU Consortium and his university received grant funding from the European Commission IMI programme for this work. Oxford University has also received grant funding from Medical Research Council, Wellcome Trust, Bill & Melinda Gates Foundation, Serum Institute of India and AstraZeneca. A.J.P. is a contributor to intellectual property licensed by Oxford University Innovation to AstraZeneca. A.J.P. is chair of the UK Joint Committee of Vaccination and Immunization and was a member of WHO SAGE until 2022. A.D.U. has been a subinvestigator for clinical trials sponsored by Pfizer and GSK, and observational studies sponsored by Sanofi, with all funds paid to her institution. A.D.U. has received funding for attending the AEP meeting in 2022. S.C. had provided clinical study advice or acted as PI for GSK, Pfizer, MedImmune, Ablynx, Gilead, Ark Biosciences, Shionogi, Janssen/Alios with fees paid to the University of Edinburgh. F.M.T. has received honoraria from GSK group of companies, Biofabri, Pfizer Inc, Sanofi Pasteur, MSD, Seqirus and Janssen for taking part in advisory boards and expert meetings and for acting as a speaker in congresses outside the scope of the submitted work. F.M.-T. has also acted as principal investigator in randomized controlled trials of the above-mentioned companies as well as Ablynx, Gilead, Regeneron, Roche, Abbott, Novavax and MedImmune, with honoraria paid to his institution. L.J.B. has regular interaction with pharmaceutical and other industrial partners. He has not received personal fees or other personal benefits. UMCU has received major funding (>€100 000 per industrial partner) for investigator-initiated studies from AbbVie, MedImmune, AstraZeneca, Sanofi, Janssen, Pfizer, MSD and MeMed Diagnostics. UMCU has received major funding for the RSV GOLD study from the Bill & Melinda Gates Foundation. UMCU has received major funding as part of the public private partnership IMI-funded RESCEU and PROMISE projects with partners GSK, Novavax, Janssen, AstraZeneca, Pfizer and Sanofi. UMCU has received major funding by Julius Clinical for participating in clinical studies sponsored by MedImmune and Pfizer. UMCU received minor funding (€1000–25 000 per industrial partner) for consultation and invited lectures by AbbVie, MedImmune, Ablynx, Bavaria Nordic, MabXience, GSK, Novavax, Pfizer, Moderna, AstraZeneca, MSD, Sanofi, Genzyme and Janssen. L.J.B. is the founding chairman of the ReSViNET Foundation. J.G.W. has been an investigator for clinical trials sponsored by pharmaceutical companies including AstraZeneca, Merck, Pfizer, Sanofi and Janssen. All funds have been paid to UMCU. J.G.W. participated in the advisory board of Janssen and Sanofi with fees paid to UMCU.

## Supplementary data

Supplementary literature review and Tables S1–S4 are available as Supplementary data at *JAC* Online.
