# Supplementary material for: Antibiotic use attributable to RSV infections during infancy—an international prospective birth cohort study
Source: J Antimicrob Chemother. 2025 May 9;80(7):1803–12. doi: 10.1093/jac/dkaf123 (PMC12209840; doi:10.1093/jac/dkaf123)
Supplement: dkaf123_Supplementary_Data [file dkaf123_supplementary_data.docx]

# **Supplementary files**

[**Supplementary files** 1](#_Toc172214041)

[Literature review of RSV-associated antibiotic use in high-income setting 2](#_Toc172214042)

[Supplementary Table 1. Overview of available literature on RSV-associated antibiotic use among young children in high-income settings 5](#_Toc172214043)

[Supplementary Table 2. RSV-associated antibiotic use by country 11](#_Toc172214044)

[Supplementary Table 3. Incidence proportion of ARI- and RSV-associated antibiotic use by site after imputation 12](#_Toc172214045)

[Supplementary Table 4. Any antibiotic use for ARI in the first year of life 13](#_Toc172214046)

[References 14](#_Toc172214047)

## **Literature review of RSV-associated antibiotic use in high-income setting**

Search strategy

To assess available evidence on RSV-associated antibiotic use in the high-income setting, we searched Pubmed on June 18^th^, 2024, using the following search terms: (RSV OR respiratory syncytial virus OR bronchiolitis) AND (antibiotics OR antibiotic OR antimicrobial) AND (infant OR infancy OR pediatric OR children), without restrictions on publication date or language.

Search terms

((RSV[Title/Abstract] OR (respiratory[Title/Abstract] AND syncytial[Title/Abstract] AND virus[Title/Abstract]) OR bronchiolitis[Title/Abstract])) AND (antibiotics[Title/Abstract] OR antibiotic[Title/Abstract] OR antimicrobial[Title/Abstract])) AND (infant[Title/Abstract] OR infancy[Title/Abstract] OR pediatric[Title/Abstract] OR children[Title/Abstract])

Inclusions criteria

- Original research
- Full-text availability
- High-income country setting
- Infant population
- Reporting on antibiotic use related to RSV ARI, RSV LRTI and/or RSV bronchiolitis

Exclusion criteria

- Reviews, guidelines, case reports
- Limited to children aged >1 year old or adults
- Study in UMIC or LMIC setting
- No laboratory-confirmed RSV diagnosis (NB: exception for outpatient setting studies)

Summary of available evidence

The initial search yielded 753 articles, of which 27 were included in this literature review. Through reference chaining, 4 additional relevant articles were identified (**Supplementary Table 1**).

*Hospital and Paediatric Intensive Care Unit (PICU) settings*

We included 18 studies reporting on antibiotic use in during hospital and PICU-admission for laboratory-confirmed RSV infections. The range of antibiotic prescriptions during RSV hospitalisations among young children varied significantly, ranging from 13.7% to 51% in high-income countries. It should be noted that the majority of these studies were conducted in tertiary centers (n=12), which likely do not reflect the typical children admitted with RSV, as they are usually admitted to general wards in secondary hospitals. Children admitted to tertiary hospitals likely have more comorbidity or are more severely ill, increasing the risk of antibiotic prescription. For children admitted to the PICU specifically, the antibiotic prescription rate was higher, ranging from 82% to 95%. Among both hospital and PICU-specific studies, only a few studies focused exclusively on infants (n=3) and on previously healthy children (n=4).

*Outpatient settings*

We included five studies on RSV-associated antibiotic use in outpatient settings. Only two were prospective cohort studies that reported data for laboratory-confirmed RSV infections in outpatient settings, whereas the remaining three studies were retrospective database analyses. Two of these three studies only reported on unspecified bronchiolitis, instead of laboratory-confirmed RSV bronchiolitis, and the third study did not include inpatient cases.

Of the two prospective studies, Rybak et al. included 1591 children with first-episode bronchiolitis attending primary care in France. Antibiotic use was 23.6% among RSV-positive children aged <2 years within 15 days after an outpatient healthcare visit, which was similar to RSV-negative children. The second, Hak (ComNet, unpublished data), in five European countries, found that antibiotics were used in 15.2% of infants with RSV acute respiratory infection (ARI) in primary care, and in 23% of RSV-positive children aged <5 years.

Of the remaining three retrospective studies, Snyder et al. reported 19.9% antibiotic use in children under 2 years with unspecified bronchiolitis in general practice and emergency departments (EDs) in the US. This was generally comparable to another US study that showed 26.3% of children under 2 years with unspecified bronchiolitis in EDs received antibiotics. Jones et al. (2023) found a 32% antibiotic use rate in RSV-positive children <18 years, yet this rate was not stratified by outpatient and inpatient setting.

*Community-based antibiotic prescribing*

We identified three prospective cohort studies and one retrospective cohort study that reported on RSV-associated antibiotic use from a community perspective. The first, a prospective birth cohort study by Thomas et al. in Finland, followed 408 infants and found an RSV incidence of 328.4 per 1000 infants in the first year of life, with 70.9% receiving antibiotics for all-severity RSV infections. They estimated that approximately 25% of all infants would receive at least one antibiotic course due to RSV by their first birthday. Another study from Finland, Toivonen et al., followed 923 newborns, and reported that 11.4% had at least one RSV-associated antibiotic treatment in the first two years of life. Antibiotics were used in 35.3% of all-severity RSV infections and accounted for 30% of antibiotic treatments for all ARIs. A third Finnish study, Heikkinen et al., found that 66% of RSV-positive children <3 years received antibiotics during any-severity RSV infections. Among infants, this rate was remarkably higher, with 91% of RSV-positive infants under one year receiving antibiotics. Finally, Abreo et al. conducted a retrospective population-based cohort study in the USA with 123,301 healthy infants. Infants with RSV bronchiolitis under six months old were more likely to have pneumonia (3.4% vs. 2.5%), otitis media (42.4% vs. 35.6%), and antibiotic fills (77.6% vs. 74.2%) compared to those without RSV. This corresponded to an odds ratio of 1.21 and an adjusted odds ratio of 1.24 for antibiotic fills. There was no dose-dependent relationship observed for antibiotic use.

Two population-based modelling studies estimated community-based rates of antibiotic prescription attributable to RSV, by linking national viral surveillance to antibiotic dispensing data. The first, Fitzpatrick et al., estimated that 5.2% of all primary care antibiotic prescriptions to Scottish infants were attributable to RSV, which corresponds to ~2.3 RSV-associated antibiotic prescriptions per 1000 infant-months^1^, which is twice as high as our estimate. RSV consistently accounted for the highest proportion of prescribed antibiotics, compared to influenza and human metapneumovirus (HMPV), including among older children (aged 1-4 years) and children with chronic conditions. A second modelling study, Taylor et al., from the UK, estimated RSV-attributable fraction of antibiotic use to be considerably higher, with 8.3% of children under 6 months of age and 11.9% aged 6-23 months receiving antibiotics due to RSV infection annually. As such, they estimated that 19.7% of antibiotics prescribed among infants aged <6 months and 14.6% in those aged 6-23 months in primary care were attributable to RSV. However, the authors recognise they may have overestimated the RSV-attributable fraction due to a lack of adjustment for seasonal confounders, such as other winter pathogens.

*Efficacy of RSV immunisation on reducing antibiotic use*

Recent clinical trials highlight the potential of preventive strategies to reduce antibiotic use in RSV cases. Trials evaluating the efficacy of RSV vaccines and monoclonal antibodies demonstrate significant reductions in antibiotic prescriptions among immunised infants compared to placebo groups. In the maternal RSVPreF trial, there was a significant reduction in antibiotic prescriptions within 90 days and 365 days after birth for infants. For infants in high-income settings, vaccine efficacy was estimated at 20.2% against all antimicrobial use, and 49.4% against lower respiratory tract infection (LRTI)-related antimicrobials within 90 days. In an analysis up to 365 days, vaccine efficacy was of 5.2% against all antimicrobial use, and 13.2% against LRTI-related antimicrobial use. Pooled analyses of the nirsevimab phase IIb and phase III trials, including late-preterm infants and those with comorbidity, showed a reduction of 23.6% in all-cause antibiotic use within 150 days of administration. To our knowledge, no real-world evidence is yet available to confirm the impact of RSV immunisation on antibiotic use during infancy.

## **Supplementary Table 1. Overview of available literature on RSV-associated antibiotic use among young children in high-income settings**

| Nr | Study | Study Design | Study Period | Country | Setting | | Population | Findings | |
| --- | --- | --- | --- | --- | --- | --- | --- | --- | --- |
| Hospital setting | | | | | | | | | |
| 1 | Van Houten 2018^2^ | Prospective cohort | 2013-2016 | Netherlands and Israel | Hospital | N=188,  RSV-positive children >1 month-18 years | | | Antibiotic use in **49%,** one-third unnecessary |
| 2 | Garciá-Garciá 2023^3^ | Prospective cohort | 2004-2022 | Spain | Hospital (secondary) | N=1715,  Children <2 years with bronchiolitis | | | Antibiotic use in **13.7%** of children admitted with RSV bronchiolitis |
| 3 | Obolski 2021^4^ | Retrospective | 2008-2018 | Israel | Hospital | N=1016,  Infants with RSV without bacterial coinfection | | | Antibiotic use in **33.4%,** classified as misuse |
| 4 | Akhras 2010^5^ | Retrospective | 2006-2007 | USA | Hospital (tertiary) | N=256,  Children <18 years with ARI | | | Antibiotic use in **51%** of RSV patients compared to **81.2%** in hMPV patients |
| 5 | Montero 2023^6^ | Retrospective | 2010-2017 | Belgium | Hospital (tertiary) | N=406,  Children <15 years with hMPV or RSV infection | | | Antibiotic use in **43%** of children with RSV |
| 6 | Hartmann 2022^7^ | Retrospective | 2015-2018 | Germany | Hospital (tertiary) | N=1312,  Children <6 years with RSV | | | Antibiotic use in **43.6%** |
| 7 | Kurz 2024^8^ | Retrospective | 2019-2022 | Switzerland | Hospital (secondary/tertiary) | N=1205,  Children with RSV or influenza | | | Antibiotic use differed: **18%** and **26%** of RSV-positive children at different sites |
| 8 | Knapper 2022^9^ | Retrospective | 2019-2020 | UK | Hospital (tertiary) | N=487,  Children and adults with RSV | | | Antibiotic use in **35%** of children |
| 9 | Cebey-López 2016^10^ | Prospective | 2011-2013 | Spain | Hospital (tertiary) | N=66,  **Previously healthy children** with RSV aged <2 years (preterm) | | | Antibiotic use in **50%** of hospitalised children |
| 10 | Kalil 2019^11^ | Retrospective | 2011-2026 | Canada | Hospital (tertiary) | N=398,  **Previously healthy children <24 months** with RSV bronchiolitis | | | Antibiotic use in **47.1%** before stewardship program, **32.0%** after |
| 11 | Tsolia 2002^12^ | Retrospective | 1997-2000 | Greece | Hospital | N=636,  Infants <1 year with bronchiolitis | | | Antibiotic use in **33%** of infants with RSV bronchiolitis |
| 12 | Papan 2020^13^ | Retrospective | 2014-2018 | Germany | Hospital | N=573,  Children <2 years with RSV or influenza | | | Antibiotic use in **28.6%** of children with RSV |
| 13 | Schreiner 2019^14^ | Retrospective | 2008-2013 | Germany | Hospital (tertiary) | N=2464,  Children with respiratory infection | | | Antibiotic use in **44.4%** of children with RSV |
| PICU-specific setting | | | | | | | | | |
| 14 | Van Woensel 2001^15^ | Retrospective | 1992-2000 | Netherlands | PICU | N=148,  Children admitted to PICU with RSV-LRTI | | | Antibiotic use in **85.1%** of children admitted to PICU with RSV-LRTI |
| 15 | Shein 2019^16^ | Retrospective | 2012-2016 | USA | PICU | N=2107,  Previously healthy children <2 years with RSV LRTI and mechanical ventilation | | | Antibiotic use in **82%** within first 2 days, 95% during hospitalisation |
| 16 | Kadmon 2020^17^ | Retrospective chart review | Not specified | Israel | PICU | N=276,  RSV-infected infants <1 year | | | Antibiotic use in **82.6%** of infants admitted with RSV |
| 17 | Thorburn 2006^18^ | Prospective cohort | 2002-2005 | UK | PICU | N=181,  Children with RSV bronchiolitis, all ages | | | Antibiotic use in **48%** before PICU admission, **96%** (158/165) during hospitalisation |
| 18 | Randolph 2004^19^ | Retrospective chart review | 1992-2002 | USA | PICU | N=165,  Previously healthy term children <36 months with RSV | | | Antibiotic use in **87.9%** of RSV-positive children |
| Outpatient settings | | | | | | | | | |
| 19 | Snyder 2021^20^ | Retrospective database analysis | 2006-2015 | USA | Outpatient (GP and ED) | N=Not specified,  Children 0-18 years with unspecified bronchitis/bronchiolitis in general practice and EDs | | | Antibiotic use in **19.9%** of children <2 years with unspecified bronchiolitis |
| 20 | Rybak 2024^21^ | Prospective test-negative cohort study | 2021-2023 | France | Outpatient | N=1591,  Children <2 years with first episode RSV bronchiolitis attending ambulatory care | | | Antibiotic use in **23.6%** of RSV-positive children within 15 days, similar to RSV-negative children |
| 21 | Jones 2023^22^ | Retrospective database analysis | 2016-2019 | USA | Outpatient ED and hospital | N=1355,  Children <18 years with viral PCR positive for RSV, HRV/ENT and/or hMPV attending outpatient clinics, ED or inpatient | | | Antibiotic use in **32%** of RSV-positive children across all settings |
| 22 | Papenburg 2019^23^ | Retrospective analysis of national survey data | 2007-2015 | USA | ED | N=612  Children <2 years with unspecified bronchiolitis (not RSV specific) presenting at ED | | | Antibiotic use in **26.3**% of children with unspecified bronchiolitis at ED who were not subsequently hospitalised |
| 23 | Hak, 2024^24^ | Prospective cohort | 2021-2023 | Spain, Italy, Belgium, UK, Netherlands | Outpatient | N=878  Children <5 years attending primary care with laboratory-confirmed RSV ARI | | | **NB: unpublished data**  Antibiotics were used in 15.2% (60/396) of laboratory-confirmed RSV ARIs among infants attending primary care, and 23% of RSV-positive children aged <5 years |
| Community settings | | | | | | | | | |
| 24 | Thomas 2021^25^ | Prospective birth cohort study | 2017-2018 | Finland | Community | N=408, newborn infants without major congenital defect or serious chronic illness (prematurity allowed) | | | RSV incidence of 328.4 per 1000 infants in first year of life. Antibiotic use in **70.9%** of all-severity RSV infection**.** The authors estimate that assuming one-third of infants acquire an RSV illness during their first year of life and three-quarters of those receive antibiotic treatment for AOM, approximately **25% of all infants will get ≥1 course of antibiotics due to RSV alone by their first birthday.** |
| 25 | Abreo 2020^26^ | Retrospective, population-based cohort study | 1995-2007 | USA | Community | N=123,301,  Healthy infants >34 GA | | | Infants with RSV bronchiolitis <6 months were more likely to have pneumonia (**3.4% and 2.5%),** otitis media (**42.4% vs. 35.6%**) and antibiotic fill **(77.6% vs. 74.2%),** corresponding to an OR: 1.21 and aOR 1.24 in the second six months of life. No dose-dependent relationship for antibiotic use |
| 26 | Toivonen et al. 2020^27^ | Prospective birth cohort study | 2008-2010 | Finland | Community | N=923,  Newborn infants, no exclusion criteria | | | During the first two years of life, **11.4%** (94/824) of all children had at least 1 RSV-associated AB treatment during follow up.  **35.3%** anitbiotic use during any-severity RSV infection  **30%** of antibiotic treatments for ARIs were prescribed for RSV infections |
| 27 | Heikkinen 2017^28^ | Prospective cohort study | Not specified | Finland | Community | N=2231  All children <14 years, regardless of any medical conditions | | | Among RSV-positive children <3 years, **66%** received AB during any-severity RSV infections  Among RSV-positive infants (<1 year), 10/11 (**91%**) received AB during any-severity RSV infections |
| Modelling studies | | | | | | | | | |
| 28 | Fitzpatrick 2021^1^ | Time-series regression model | 2009-2017 | Scotland | Not specified | Children in their first year of life | | | **5.2%** of antibiotics in first year of life attributable to RSV |
| 29 | Taylor 2016^29^ | Time-series regression modelling | 1995-2009 | UK | Not specified | Children 0-17 years | | | **8.3%** of children <6 months and **11.9%** of 6-23 months received antibiotics for RSV-attributed infection |
| Clinical trials | | | | | | | | | |
| 30 | Simoes 2023^30^ | Multicenter, double-blind RCT | 2018-2020 | 21 countries in Europe, North America and Asia, South Africa | RCT | N=1490,  Term and preterm infants, including those with comorbidity | | | All-cause antibiotic use was reduced by **23.6%** (3.8-29.3) in 150 days after nirsevimab administration |
| 31 | Lewnard 2022^31^ | Multicenter, double-blind RCT | 2016-2019 | Argentina, Australia, Chile, Bangladesh, Mexico, New Zealand, Philippines, South Africa, Spain, UK, US | RCT | N=2978  Live-born infants to mothers who received either RSVPreF vaccine or placebo at between 28-36 weeks of gestation | | | Vaccine efficacy in HIC of **20.2%** for all antimicrobials, and **49.4%** for LRTI antimicrobials within 90 days = maternal vaccination prevented **3.6 antimicrobial prescription courses for every 100 infants**  Vaccine efficacy in HIC of **5.2%** for all antimicrobials, and **13.2%** for LRTI-related antimicrobials to end of follow-up (365 days) |

ARI: acute respiratory infection; LRTI: lower respiratory tract infection; RSV: respiratory syncytial virus; HIC: high-income countries

## **Supplementary Table 2. RSV-associated antibiotic use by country**

|  | **Scotland** | **England** | **Spain** | **Finland** | **The Netherlands** |
| --- | --- | --- | --- | --- | --- |
| **RSV hospitalisations (total cohort)** | | | | | |
| **Antibiotic use** |  |  |  |  |  |
| All RSV hospitalisations | 4/48 (8.3%) | 9/28 (32.1%) | 10/25 (40.0%) | 8/21 (38.1%) | 2/23 (8.7%) |
| General ward/HDU | 3/46 (6.5%) | 8/27 (29.6%) | 8/22 (36.4%) | 7/20 (35.0%) | 2/22 (9.1%) |
| ICU | 1/2 (50.0%) | 1/1 (100%) | 2/3 (66.7%) | 1/1 (100%) | 0/1 (0%) |
| **RSV ARI (active surveillance cohort)** | | | | | |
| **Antibiotic use** |  |  |  |  |  |
| All RSV ARI | 1/53 (1.9%) | 0/31 (0.0%) | 5/60 (8.3%) | 6/20 (30%) | 1/85 (1.1%) |
| Medically attended | 1/23 (4.3%) | 0/18 (0.0%) | 5/34 (14.7%) | 6/14 (42.9%) | 1/41 (2.4%) |
| Outpatient | 1/22 (4.5%) | 0/14 (0.0%) | 4/28 (14.3%) | 5/13 (38.5%) | 1/36 (2.8%) |
| Hospitalised | 0/1 (0.0%) | 0/4 (0.0%) | 1/6 (16.7%) | 1/1 (100%) | 0/6 (0.0%) |

HDU: high-dependency unit; ICU: intensive care unit

## **Supplementary Table 3. Incidence proportion of ARI- and RSV-associated antibiotic use by site after imputation**

|  | **Scotland (n=203)** | **England (n=198)** | **Spain (n=205)** | **Finland (n=200)** | **The Netherlands (n=187)** |
| --- | --- | --- | --- | --- | --- |
| **All ARI-associated antibiotic use** |  |  |  |  |  |
| Incidence proportion (95%CI) | 1.1%  (0.4-3.3) | 3.6%  (2.0-6.6) | 8.6%  (5.9-12.6) | 9.3%  (6.4-13.3) | 3.8%  (2.1-6.9) |
| **RSV-positive ARI-associated antibiotic use** |  |  |  |  |  |
| Incidence proportion  (95%CI) | 0.5%  (0.1-2.2) | 0.1%  (0.3-3.0) | 2.6%  (1.3-5.2) | 2.6%  (1.3-5.2) | 0.5%  (0.1-2.4) |
| **RSV-negative ARI-associated antibiotic use** |  |  |  |  |  |
| Incidence proportion  (95%CI) | 0.6%  (0.2-2.7) | 3.5%  (1.9-6.5) | 6.1%  (3.8-9.5) | 6.7%  (4.3-10.2) | 3.8%  (2.3-5.0) |

Incidence proportion was defined as the proportion of infants who used antibiotics during RSV ARI at least once during the first year of life, among all infants in the nested cohort. Incidence rate was defined as number of RSV-associated antibiotic prescriptions per 1000 infant-months of follow-up. Both measures were calculated after multiple imputation of missing values for RSV status, medical attendance, and antibiotic use.

## **Supplementary Table 4. Any antibiotic use for ARI in the first year of life**

|  | **Infants hospitalised**  **with ARI in first year of life^1^** | | |  | **Infants not hospitalised**  **with ARI in first year of life** | |
| --- | --- | --- | --- | --- | --- | --- |
|  | **RSV hospitalisation (N=139)** | **RSV-negative hospitalisation (N=149)** | **p-value^2^** | **Not-hospitalised (N=8650)** | | ***p*-value^3^** |
| ARI-associated antibiotic use in first year of life, excluding antibiotics use during ARI hospitalisation | 28.7% (33/115) | 38.5% (50/130) | 0.11 | 15.2% (1168/7679) | | <0.001 |
| 1 course | 57.6% (19/33) | 59.6% (28/47) |  | 67.1% (777/1158) | |  |
| 2 courses | 21.2% (7/33) | 27.7% (13/47) |  | 21.6% (250/1158) | |  |
| 3 courses | 6.1% (2/33) | 2.1% (1/47) |  | 6.0% (69/1158) | |  |
| ≥4 courses | 15.2% (5/30) | 10.6% (5/47) |  | 5.4% (62/1158) | |  |

ARI; acute respiratory infection.

Antibiotic use as reported by parents at infant’s first birthday. Infants who were hospitalised but with at least one hospitalisation with unknown RSV status were excluded (n=40), since we cannot exclude that these infants had been hospitalised with RSV.

^1^ Infants hospitalised with ARI were exclusively categorised in 1). At least one RSV-associated hospitalisation 2). Hospitalised with non-RSV ARI. 12 infants had both an RSV-positive and an RSV-negative hospitalisation and were only included in the RSV-positive group. In case of hospitalisation with unknown RSV status, infants were excluded (n=40/9154; 0.4%).

^2^ Comparison between infants hospitalised with RSV and other ARI.

^3^ Comparison between infants hospitalised with RSV and infants not hospitalised with ARI in the first year of life.

## **Supplementary Methods: RSV testing procedures**

At all sites, a nasal sample was collected by a trained member of the study team during a home visit for each ARI episode by using minitip flocked swabs (FLOQSwab, Copan Diagnostics, California, USA), using a standardized protocol. Swabs were directly stored in viral transport medium (MicroTest M4RT [Remel, 3 mL]). All samples were stored at –80°C. After the end of the study, all samples were tested with in-house RSV quantitative reverse transcription PCR (RT-qPCR; appendix p 2). In addition, a point of care test (POCT, Alere i RSV assay [Alere, Waltham, MA, USA]) was performed at the time of sample collection at the three sites in Spain, England, and the Netherlands. If the infant had an RSV-positive ARI episode, POCT was not performed during further ARIs. Staff received get hands-on training on participant sampling and how to perform the Alere i RSV POCT according to the manufacturer’s instructions. In short, 200 μL of the viral transport medium mixed with the swab will be aspirated with the included transfer pipette and added to the sample receiver liquid (elution buffer) and mixed for 10 seconds. All personnel received hands-on training to ensure consistent sample collection and POCT performance, minimizing variability.

**References**

1 Fitzpatrick T, Malcolm W, McMenamin J, Reynolds A, Guttmann A, Hardelid P. Community-Based Antibiotic Prescribing Attributable to Respiratory Syncytial Virus and Other Common Respiratory Viruses in Young Children: A Population-Based Time-series Study of Scottish Children. Clinical Infectious Diseases 2021; 72: 2144–53.

2 Van Houten CB, Naaktgeboren C, Buiteman BJM, et al. Antibiotic Overuse in Children with Respiratory Syncytial Virus Lower Respiratory Tract Infection. Pediatric Infectious Disease Journal 2018; 37: 1077–81.

3 García-García ML, Alcolea S, Alonso-López P, et al. Antibiotic Utilization in Hospitalized Children with Bronchiolitis: A Prospective Study Investigating Clinical and Epidemiological Characteristics at a Secondary Hospital in Madrid (2004–2022). Pathogens 2023; 12. DOI:10.3390/pathogens12121397.

4 Obolski U, Kassem E, Na’amnih W, Tannous S, Kagan V, Muhsen K. Unnecessary antibiotic treatment of children hospitalised with respiratory syncytial virus (RSV) bronchiolitis: risk factors and prescription patterns. J Glob Antimicrob Resist 2021; 27: 303–8.

5 Akhras N, Weinberg JB, Newton D. Human metapneumovirus and respiratory syncytial virus: Subtle differences but comparable severity. Infect Dis Rep 2010; 2: 35–9.

6 Illan Montero J, Berger A, Levy J, Busson L, Hainaut M, Goetghebuer T. Retrospective comparison of respiratory syncytial virus and metapneumovirus clinical presentation in hospitalized children. Pediatr Pulmonol 2023; 58: 222–9.

7 Hartmann K, Liese JG, Kemmling D, et al. Clinical Burden of Respiratory Syncytial Virus in Hospitalized Children Aged ≤5 Years (INSPIRE Study). Journal of Infectious Diseases 2022; 226: 386–95.

8 Kurz H, Sever-Yildiz G, Kocsisek C V., et al. Respiratory Syncytial Virus and Influenza during the COVID-19 Pandemic: A Two-center Experience. Pediatric Infectious Disease Journal 2024; 43: 410–4.

9 Knapper F, Ellis J, Bernatoniene J, Williams P. The Burden of Respiratory Syncytial Virus Disease in Children and Adults Hospitalized in a Large Tertiary Hospital in the United Kingdom: A Retrospective Study. Pediatric Infectious Disease Journal 2022; 41: E541–3.

10 Cebey-López M, Pardo-Seco J, Gómez-Carballa A, et al. Bacteremia in children hospitalized with respiratory syncytial virus infection. PLoS One 2016; 11. DOI:10.1371/journal.pone.0146599.

11 Kalil J, Bowes J, Reddy D, Barrowman N, Le Saux N. Pediatric Inpatient Antimicrobial Stewardship Program Safely Reduces Antibiotic Use in Patients with Bronchiolitis Caused by Respiratory Syncytial Virus: A Retrospective Chart Review. Pediatr Qual Saf 2019; 4: e211.

12 Tsolia MN, Kafetzis D, Danelatou K, et al. Epidemiology of respiratory syncytial virus bronchiolitis in hospitalized infants in Greece. Eur J Epidemiol 2002; 18: 55–61.

13 Papan C, Willersinn M, Weiß C, Karremann M, Schroten H, Tenenbaum T. Antibiotic utilization in hospitalized children under 2 years of age with influenza or respiratory syncytial virus infection - A comparative, retrospective analysis. BMC Infect Dis 2020; 20. DOI:10.1186/s12879-020-05336-5.

14 Schreiner D, Groendahl B, Puppe W, et al. High antibiotic prescription rates in hospitalized children with human metapneumovirus infection in comparison to RSV infection emphasize the value of point-of-care diagnostics. Infection 2019; 47: 201–7.

15 Van Woensel JBM, Von Rosenstiel IA, Kimpen JLL, Spanjaard L, Van Aalderen WMC. Antibiotic use in pediatric intensive care patients with lower respiratory tract infection due to respiratory syncytial virus [8]. Intensive Care Med. 2001; 27: 1436.

16 Shein SL, Kong M, McKee B, O’Riordan M, Toltzis P, Randolph AG. Antibiotic Prescription in Young Children With Respiratory Syncytial Virus–Associated Respiratory Failure and Associated Outcomes. Pediatric Critical Care Medicine 2019; 20: 101–9.

17 Kadmon G, Feinstein Y, Lazar I, et al. Variability of Care of Infants With Severe Respiratory Syncytial Virus Bronchiolitis. Pediatric Infectious Disease Journal 2020; 39: 808–13.

18 Thorburn K, Harigopal S, Reddy V, Taylor N, Van Saene HKF. High incidence of pulmonary bacterial co-infection in children with severe respiratory syncytial virus (RSV) bronchiolitis. Thorax 2006; 61: 611–5.

19 Randolph AG, Reder L, Englund JA. Risk of Bacterial Infection in Previously Healthy Respiratory Syncytial Virus-Infected Young Children Admitted to the Intensive Care Unit. Pediatr Infect Dis J 2004; 23: 990–4.

20 Snyder RL, King LM, Hersh AL, Fleming-Dutra KE. Unnecessary antibiotic prescribing in pediatric ambulatory care visits for bronchitis and bronchiolitis in the United States, 2006-2015. Infect Control Hosp Epidemiol 2021; 42: 612–5.

21 Rybak A, Cohen R, Bangert M, et al. Assessing the Burden of Respiratory Syncytial Virus-related Bronchiolitis in Primary Care and at 15-Day and 6-Month Follow-up Before Prophylaxis in France: A Test-negative Study. Pediatric Infectious Disease Journal 2024; 43: 657–62.

22 Jones MU, Montgomery AS, Coskun JD, Marcelo RZ, Sutton AB, Raiciulescu S. Comparing the Clinical Courses of Children with Human Rhinovirus/Enterovirus to Children with Other Respiratory Viruses in the Outpatient Setting. Pediatric Infectious Disease Journal 2023; 42: E432–9.

23 Papenburg J, Fontela PS, Freitas RR, Burstein B. Inappropriate Antibiotic Prescribing for Acute Bronchiolitis in US Emergency Departments, 2007–2015. J Pediatric Infect Dis Soc 2019; 8: 567–70.

24 Hak S, Sankatsing V, Wildenbeest J, et al. Disease burden of RSV infections and associated healthcare use among young children in primary care in five European countries. ESPID 2024 Conference, Copenhagen. 2024; published online May. https://congress.sanofimedical.com/espid-2024/respiratory-syncytial-virus-infection-%28rsv%29/epidemiology-burden/7273/7269 (accessed June 12, 2024).

25 Thomas E, Mattila JM, Lehtinen P, Vuorinen T, Waris M, Heikkinen T. Burden of Respiratory Syncytial Virus Infection during the First Year of Life. J Infect Dis 2021; 223: 811–7.

26 Abreo A, Wu P, Donovan BM, et al. Infant Respiratory Syncytial Virus Bronchiolitis and Subsequent Risk of Pneumonia, Otitis Media, and Antibiotic Utilization. Clinical Infectious Diseases 2020; 71: 211–4.

27 Toivonen L, Karppinen S, Schuez-Havupalo L, et al. Respiratory syncytial virus infections in children 0-24 months of age in the community. J Infect 2020; 80: 69–75.

28 Heikkinen T, Ojala E, Waris M. Clinical and Socioeconomic Burden of Respiratory Syncytial Virus Infection in Children. J Infect Dis 2017; 215: 17–23.

29 Taylor S, Taylor RJ, Lustig RL, et al. Modelling estimates of the burden of respiratory syncytial virus infection in children in the UK. BMJ Open 2016; 6. DOI:10.1136/bmjopen-2015-009337.

30 Simões EAF, Madhi SA, Muller WJ, et al. Efficacy of nirsevimab against respiratory syncytial virus lower respiratory tract infections in preterm and term infants, and pharmacokinetic extrapolation to infants with congenital heart disease and chronic lung disease: a pooled analysis of randomised controlled trials. Lancet Child Adolesc Health 2023; 7: 180–9.

31 Lewnard JA, Fries LF, Cho I, Chen J, Laxminarayan R. Prevention of antimicrobial prescribing among infants following maternal vaccination against respiratory syncytial virus. 2022. DOI:10.1073/pnas.
